# Identification of target genes to control acetate yield during aerobic fermentation with *Saccharomyces cerevisiae*

**DOI:** 10.1186/s12934-016-0555-y

**Published:** 2016-09-15

**Authors:** José Antonio Curiel, Zoel Salvadó, Jordi Tronchoni, Pilar Morales, Alda Joao Rodrigues, Manuel Quirós, Ramón Gonzalez

**Affiliations:** 1 Consejo Superior de Investigaciones Científicas-Universidad de La Rioja-Gobierno de La Rioja, Departamento de Enología, Instituto de Ciencias de la Vid y del Vino, Finca La Grajera, Ctra. De Burgos Km. 6, 26007 Logroño, La Rioja Spain; 2 Evolva Biotech A/S, Copenhagen Ø, Denmark

**Keywords:** Aerobic fermentation, Crabtree effect, Volatile acidity, Alcohol level reduction, Carbon catabolite derepression, Wine

## Abstract

**Background:**

Aerobic fermentation of grape must, leading to respiro-fermentative metabolism of sugars, has been proposed as way of reducing alcohol content in wines. Two factors limit the usefulness of *Saccharomyces cerevisiae* for this application, the Crabtree effect, and excess volatile acidity under aerobic conditions. This work aimed to explore the impact on ethanol acetate production of different *S. cerevisiae* strains deleted for genes previously related with the Crabtree phenotype.

**Results:**

Recombinant strains were constructed on a wine industrial genetic background, FX10. All yeast strains, including FX10, showed respiro-fermentative metabolism in natural grape must under aerobic conditions, as well as a concomitant reduction in ethanol yield. This indicates that the Crabtree effect is not a major constrain for reaching relevant respiration levels in grape must. Indeed, only minor differences in ethanol yield were observed between the original and some of the recombinant strains. In contrast, some yeast strains showed a relevant reduction of acetic acid production. This was identified as a positive feature for the feasibility of alcohol level reduction by respiration. Reduced acetic acid production was confirmed by a thorough analysis of these and some additional deletion strains (involving genes *HXK2*, *PYK1*, *REG1*, *PDE2* and *PDC1*). Some recombinant yeasts showed altered production of glycerol and pyruvate derived metabolites.

**Conclusions:**

*REG1* and *PDC1* deletion strains showed a strong reduction of acetic acid yield in aerobic fermentations. Since *REG1* defective strains may be obtained by non-GMO approaches, these gene modifications show good promise to help reducing ethanol content in wines.

**Electronic supplementary material:**

The online version of this article (doi:10.1186/s12934-016-0555-y) contains supplementary material, which is available to authorized users.

## Background

Rising sugar concentration of grape berries at harvest, as a consequence of global climate change [1], has become a matter of concern for winemakers, particularly those from viticultural regions located in warm countries. A second factor contributing to increasing sugar levels in grape must is the current consumer preferences for well-structured, full body wines, which require an optimal phenolic maturity of grapes. Under standard winemaking conditions, excess sugar in grape must, together with other changes in must composition, also related to global climate warming, translates into fermentation troubles and, more significantly, into high alcohol content in the final wines. High ethanol content in wines can compromise product quality by exacerbating the perception of some mouth feel features such as hotness and viscosity. Sweetness, acidity, aroma, flavor intensity, and textural properties can also be negatively impacted, to a lesser extent [2–4]. In addition, wines with a high alcoholic degree might be rejected by some consumers on the ground of health or road safety considerations. International trade of such wines might also be hampered by significant increases in taxes, depending on the countries involved.

Currently, there is not a single approach that would completely solve this issue. Therefore, the wine industry is seeking for complementary solutions targeting different steps of the production cycle, including grapevine clonal selection, vineyard management, removal of excess sugar, adaptation of winemaking practices, using metabolic inhibitors, or partial ethanol removal [5]. Concerning the fermentation step in winemaking, most of the efforts are currently focused on the use of non-*Saccharomyces* wine yeast species/strains showing lower ethanol yields than *Saccharomyces cerevisiae* [6, 7]. Some years ago, our research group proposed taking advantage of the respiratory metabolism of yeasts as an approach for reducing ethanol yield [8]. Several yeast strains were shown to be able to reduce ethanol yields during aerated fermentation, as compared to standard conditions [9]; but many of them, including *S. cerevisiae* strains, were discarded for this application, due to a significant increase in acetic acid production under aerobic conditions [9, 10]. Nevertheless, the proposed technology requires *S. cerevisiae* to be inoculated either simultaneously or subsequently to non-*Saccharomyces* starters, in order to ensure fermentation completion. In addition, *S. cerevisiae* will be almost invariably present in the natural microbiota of grape must. Therefore, volatile acidity due to the metabolic activity of *S. cerevisiae* would always remain a matter of concern for the fermentation of wine under aerated conditions, as shown in studies using a combination of *Metschnikowia pulcherrima* and *S. cerevisiae* [11].

**Fig. 1:**
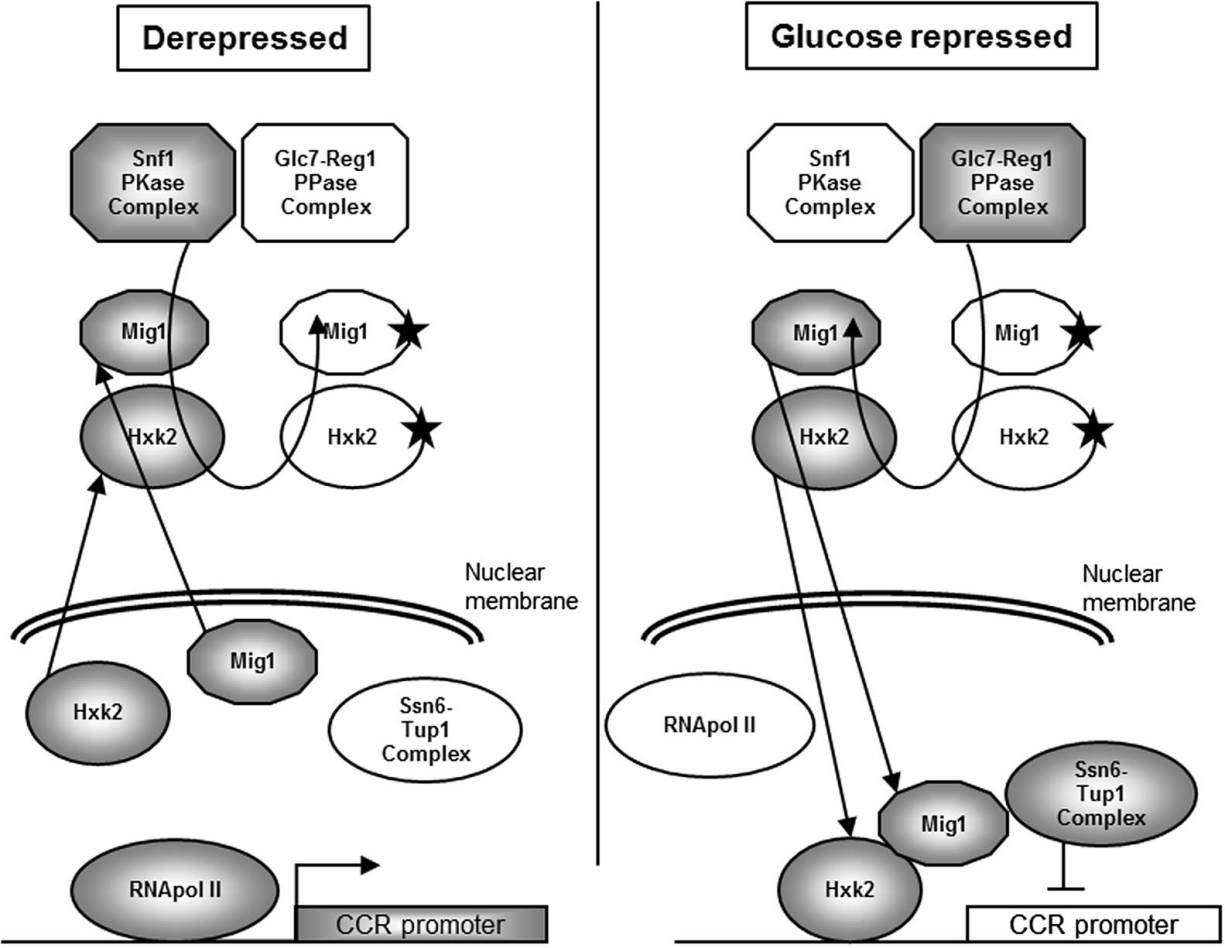
Simplified model showing the main role of Hxk2 and Reg1 in carbon catabolite repression (CCR). The different elements are shown in grey for the active state and empty for the inactive state. Stars on Mig1 and Hxk2 indicate a phosphorylated state. Several elements shown in the model have additional functions (either in CCR or not), and not all the factors involved in CCR are shown. Both Hxk2 and Reg1 must be active for efficient repression of many genes under CCR control Model based on [21], with additional information from [22]

This work started with the aim of constructing *S. cerevisiae* wine yeast strains showing an alleviated Crabtree effect, as a way to improve ethanol content reduction by respiration. This metabolic trait makes *S. cerevisiae* preferentially consume sugars by fermentation, independently of oxygen availability, and seems to be regulated at various levels, from transcriptional repression of respiratory functions [12], to kinetic features of enzymes involved in pyruvate metabolism in this species [13, 14]. Some mutations have been related to alleviated Crabtree effect in *S. cerevisiae*, including loss-of-function of *REG1* [15] or *HXK2* [16–19]; as well as reduced pyruvate kinase (Pyk1) levels [20]. *REG1* and *HXK2* are involved in carbon catabolite repression (CCR) in this species (Fig. 1). Reg1 is the regulatory subunit of the Glc7-Reg1 protein phosphatase complex, targeting it to several CCR related substrates, including Snf1 or Mig1 [21]. Hxk2 is a moonlighting protein. In addition to its hexokinase activity (it is the main cytoplasmic hexokinase during yeast growth on glucose), it participates in transcriptional repression in the nucleus, together with Mig1 [21]. Nucleocytoplasmic localization of both Hxk2 and Mig1 depends on its phosphorylation state [22]. The impact of lowered Pyk1 activity on the Crabtree effect is probably related to the rate of pyruvate accumulation [13, 14, 20] (Additional file 1: Figure S1).

In this work we found reduced ethanol yields in *S. cerevisiae* during the fermentation of natural or synthetic grape must under aerobic conditions, despite the Crabtree effect. This confirmed recent results from parallel research lines [9–11]. In contrast, we found an unexpected positive impact of some genetic modifications on volatile acidity (i.e. acetic acid), the main drawback of *S. cerevisiae* for this application [9–11].

## Results

### Selection of target genes for alcohol level reduction by respiration

We analyzed the metabolic impact of the deletion of five target genes in a *S. cerevisiae* industrial wine yeast background (Table 1). The aim was the identification of yeast genetic modification strategies that would improve alcohol level reduction during wine fermentation under aerobic conditions. Given that the Crabtree effect is the major metabolic feature of *S. cerevisiae* restricting respiratory metabolism, three of these genes were selected according to published information about the impact of gene deletions on respiro-fermentative metabolism. Herwig and von Stockar [15] found that mutant yeast strains defective for either *HXK2* or *REG1* alleviated repression of respirative functions by external glucose. Several other authors have reported reduced formation of fermentation products, as well as higher biomass yield, by yeast strains carrying inactive alleles of *HXK2* [16–19]. On the other side, Pearce et al. [20] described recombinant yeast strains with reduced pyruvate kinase (Pyk1) levels, which showed increased relative flux trough the TCA pathway.

**Table 1:** Yeast strains used for this study

Strains	Genotype	Source
FX10	Homozygote industrial yeast derivative	Laffort
FHXK2	FX10 hxk2::kanMX4/hxk2::kanMX4	This study
FPYK1	FX10 PYK1/pyk1::kanMX4	This study
FREG1	FX10 reg1::kanMX4/reg1::kanMX4	This study
FPDE2	FX10 pde2::kanMX4/pde2::kanMX4	This study
FPDC1	FX10 pdc1::kanMX4/pdc1::kanMX4	This study

**Fig. 2:**
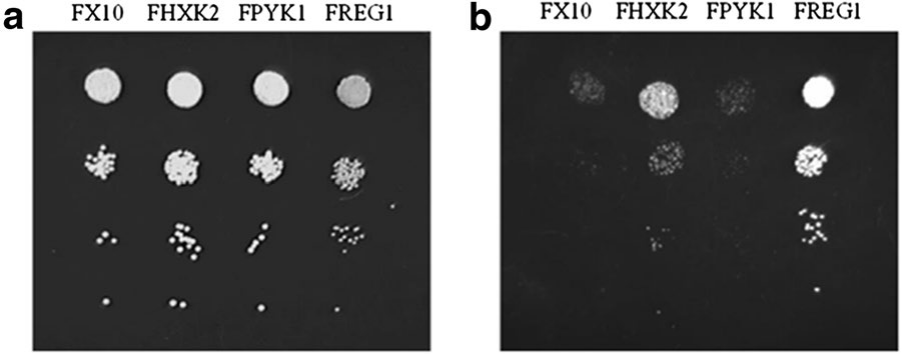
Growth of S. cerevisiae FX10 and different recombinant derivatives on YPgalactose. **a** Without 2-deoxy glucose; **b** supplemented with 2-deoxy glucose

FX10 based recombinant strains defective for each one of these three genes were constructed as described in Methods. For *HXK2* or *REG1* both alleles were deleted (Table 1). However, *PYK1* being an essential gene in *S. cerevisiae*, only one of the two alleles was deleted, in order to reduce gene dosage (Table 1). According to the functions previously described for these genes, we found that strains FREG1 and FHXK2 were defective for carbon catabolite repression, while FPYK1 was normally repressed, as expected (Fig. 2). However, preliminary analysis of ethanol yields during the fermentation of natural grape must under moderate aeration, failed to identify a relevant impact of these gene deletions on respiro-fermentative metabolism. Interestingly, some of the recombinant strains showed reduced acetic acid production as compared to FX10 (data not shown).

In order to identify additional potential targets for genetic improvement of aerobic metabolism, with the aim of reducing alcohol levels, we performed a competition experiment of the homozygous deletion collection. The continuous cultivation described by Herwig and von Stockar [15] was modified by running in synthetic medium (D = 0.18 h^−1^), with 10 g/L glucose and full aeration for 12 generations. Results of the competition experiment were analyzed as described [23]. Five gene deletions, showing improved growth according to this study, were tested for the ethanol and acetate yields during the fermentation of grape must under aerobic conditions. While none of the strains showed reduced ethanol yield, the strain deleted for *PDE2* showed a clear reduction in acetic acid yield.

We could draw three main observations from our preliminary analysis. First, despite the Crabtree effect, the wine yeast strain FX10 was able to respire a significant amount of sugar under aerobic conditions. Second, as previously shown [9–11, 24–26], increased acetic acid production under aerobic conditions is a chief limitation in order to use *S. cerevisiae* for alcohol level reduction. Finally, the main advantage observed for some of the recombinant strains tested above was, indeed, reduced acetic acid yields under aerobic conditions. For this reason, we decided to focus on the reduction of aerobic acetic acid yields as the main target for wine yeast improvement. This would allow alcohol level reduction without the drawback of excess volatile acidity. According to this new focus, we included an additional gene in the study, *PDC1* (Additional file 1: Figure S1) Pyruvate decarboxylase (encoded by *PDC1*) is a key enzyme in alcoholic fermentation, catalyzing the decarboxylation of pyruvate to acetaldehyde, as an intermediary step towards ethanol production [27]. Acetaldehyde can also be oxidized to acetic acid, and several authors have found decreased acetate production by Pdc1 defective yeast strains [28]. Hence, *PDE2* and *PDC1* deletions were introduced in the FX10 genetic background, in order to perform a comparative characterization (Table 1).

### Main fermentation products

Characterization of the five recombinant strains mentioned above was performed in natural white grape must under aerobic conditions (as well as under anaerobic conditions for comparison purposes). Only one of the gene deletions assayed resulted in a severe impairment of yeast growth, *PDE2*. Final biomass values for FPDE2 were about one-half those of the control strains, for anaerobic or aerobic conditions respectively (Table 2). The higher biomass production observed for all the strains under aerobic conditions (Table 2) is in agreement with a significant portion of sugar being consumed by respiratory metabolism. Concerning residual sugar, data for FREG1 indicate *REG1* deletion is detrimental for yeast metabolism; despite no impact on cell numbers was observed. FREG1 was the only strain leaving some residual sugar after seven days of culture, under either aerobic (38 g/L) or anaerobic (22 g/L) conditions (Table 2). This was in agreement with results from chemostat cultures mentioned above. Residual sugar was almost exclusively constituted by fructose (Additional file 2: Figure S2). Indeed, FREG1was the only strain showing an altered preference for the two monosaccharides present in grape must (Additional file 2: Figure S2).

**Table 2:** Main metabolites in fermentations run by the parent FX10 strain and recombinant derivatives

	^1^Biomass (DO_600_ nm)		^2^Residual sugars (g/L)		^2^Ethanol (% vol/vol)		^2^Acetic acid (g/L)		^2^Glycerol (g/L)	
	Anaerobic	Aerobic	Anaerobic	Aerobic	Anaerobic	Aerobic	Anaerobic	Aerobic	Anaerobic	Aerobic
FX10	13.93 ± 1.93^b^	20.93 ± 1.53^bc^	0.10 ± 0.17^a^	0.03 ± 0.06^a^	8.70 ± 0.24^c^	5.63 ± 0.35^b^	0.14 ± 0.03^bc^	3.34 ± 0.48^cd^	10.28 ± 0.24^a^	5.80 ± 0.20^a^
FHXK2	14.53 ± 1.52^b^	22.70 ± 2.35^bc^	0.10 ± 0.17^a^	0.03 ± 0.06^a^	8.71 ± 0.34^c^	5.59 ± 1.01^b^	0.16 ± 0.08^c^	3.57 ± 0.38^d^	11.00 ± 0.17 ^ab^	5.98 ± 0.03^a^
FPYK1	10.63 ± 1.67^ab^	24.63 ± 1.38^c^	0.17 ± 0.21^a^	0.06 ± 0.06^a^	8.51 ± 0.09^c^	5.79 ± 0.21^b^	0.11 ± 0.03^abc^	4.57 ± 0.89^d^	10.52 ± 0.03^ab^	6.10 ± 0.20^a^
FREG1	12.30 ± 0.70^ab^	24.72 ± 2.02^c^	22.50 ± 5.11^b^	38.00 ± 2.17^b^	6.70 ± 0.40^a^	4.03 ± 0.34^a^	0.15 ± 0.04^c^	0.15 ± 0.02^a^	10.73 ± 1.47^ab^	17.57 ± 0.32^b^
FPDC1	11.71 ± 2.78^ab^	18.75 ± 2.19^b^	ND	ND	8.19 ± 0.26^bc^	5.64 ± 0.37^b^	0.06 ± 0.04^ab^	0.73 ± 0.47^ab^	9.85 ± 0.06^a^	5.55 ± 0.44^a^
FPDE2	7.53 ± 2.05^a^	13.13 ± 1.95^a^	0.07 ± 0.12^a^	ND	7.59 ± 0.25^b^	6.49 ± 0.71^b^	0.04 ± 0.03^a^	1.91 ± 0.76^bc^	12.43 ± 0.91^b^	6.43 ± 0.67^a^

Values are shown as mean ± SD of three biological replicates. The glucose and fructose contents of the natural must in the fermentations were ranged in 190.4–195.8 g/L. Different letters indicate statistically significant differences (HSD Tukey) for values in the same column

*ND* not detectable

^1^Analyses were performed after 4 days of fermentation

^2^Analyses were performed after 7 days of fermentation

Comparison of ethanol yields on sugar between anaerobic and aerobic conditions (Table 3) confirmed that, despite the Crabtree effect, even wild type strains of *S. cerevisiae* showed a great deal of respiratory metabolism in grape must under aerobic conditions. FREG1 and FPDE2 show the extreme values (0.21 and 0.27 g/g, respectively) of ethanol yield under aerobic conditions; while the ethanol yield for FREG1 was also the lowest one (0.31 g/g) under anaerobic conditions. However, most of the recombinant strains showed ethanol yield values that were indistinguishable from the control FX10 strain, not only under anaerobic conditions (where no respiration can take place) but also under aerobic conditions.

**Table 3:** Yields of ethanol, acetic acid and glycerol calculated for the parent FX10 strain and recombinant derivatives

	*Y*_*E/S*_ (g/g)		*Y*_*A/S*_ (mg/g)		*Y*_*G/S*_ (mg/g)	
	Anaerobic	Aerobic	Anaerobic	Aerobic	Anaerobic	Aerobic
FX10	0.36 ± 0.01^b^	0.23 ± 0.01^ab^	0.76 ± 0.16^bc^	17.56 ± 2.30^cd^	54.04 ± 1.21^a^	30.47 ± 1.04^a^
FHXK2	0.36 ± 0.01^b^	0.23 ± 0.01^ab^	0.86 ± 0.04^bc^	18.62 ± 2.22^d^	57.27 ± 1.53^ab^	31.15 ± 0.64^a^
FPYK1	0.35 ± 0.01^b^	0.24 ± 0.01^ab^	0.59 ± 0.16^abc^	23.84 ± 4.98^d^	54.78 ± 2.16^a^	31.76 ± 1.51^a^
FREG1	0.31 ± 0.01^a^	0.21 ± 0.01^a^	0.94 ± 0.28^c^	1.01 ± 0.17^a^	63.74 ± 4.26^bc^	115.29 ± 3.42^b^
FPDC1	0.36 ± 0.02^b^	0.24 ± 0.01^ab^	0.35 ± 0.23^ab^	3.91 ± 2.44^ab^	54.27 ± 3.52^a^	29.93 ± 1.89^a^
FPDE2	0.34 ± 0.02^b^	0.27 ± 0.01^b^	0.22 ± 0.20^a^	10.04 ± 4.02^bc^	71.21 ± 2.36^c^	33.79 ± 3.50^a^

Values are shown as mean ± SD of three biological replicates. *Y*_*E/S*_ ethanol yield on sugar, *Y*_*A/S*_ acetic acid yield on sugar, *Y*_*G/S*_ glycerol yield on sugar. Different letters indicate statistically significant differences (HSD Tukey) for values in the same column

Acetic acid production under anaerobic conditions by some of the strains assayed is suitable for the production of quality wines (Table 2), considering that, above 0.8 g/L, acetic acid may confer an undesirable acidic taste and unpleasant vinegar aroma to wine [29]. FPDE2 showed the lowest acetic acid yield under anaerobic conditions (Table 3). On the other side, the trend towards increased acetic acid production under aerobic conditions that was previously described [9, 11, 25] was confirmed for this set of yeast strains. Indeed, acetic acid production under aerobic conditions was unacceptably high for most of the strains, apart from FREG1 and FPDC1 (Table 2). Actually, FREG1 seems to be an exception to the general rule of increasing acetic acid yield under aerobiosis. While the other yeast strains experienced an increase in acetic acid yield, ranging from 8 to 40 times under aerobic as compared to anaerobic conditions, acetic acid yield for FREG1 was similar under both culture conditions.

Quantitative differences in glycerol yields in anaerobiosis were relatively small (as compared for example with differences in acetic acid yields), even though FREG1 and FPDE2 showed higher (statistically significant) glycerol yields than the control strain (Table 3). Aeration resulted in a reduction of about one half in glycerol yield for most of the strains. However, FREG1 showed the opposite behavior, with twice as much glycerol yield under aerobic conditions as compared to anaerobiosis (Table 3). The final glycerol content of aerobic FREG1 fermentation is indeed in the upper part of the normal range accepted for quality wines (12–18 mg/L; Table 2).

### Other pyruvate derived metabolites

In view of the striking differences in acetic acid yields shown by the recombinant strains (Table 3), we wondered whether other metabolic by-products from the pyruvate node (acetaldehyde, acetoin and 2,3 butanediol) were also affected by the gene deletions tested. No statistically significant differences were found for the yeast strains concerning acetaldehyde production under anaerobic conditions (Table 4). The general trend for acetaldehyde levels was towards higher values in aerobiosis, with the exception of FREG1. Deletion of *PDC1* results in a huge increase in acetaldehyde production under aerobic conditions (Table 4).

**Table 4:** Volatile metabolites produced by the parent FX10 strain and recombinant derivatives

	Acetaldehyde		Acetoin		2,3 butanediol	
	Anaerobic	Aerobic	Anaerobic	Aerobic	Anaerobic	Aerobic
FX10	0.094 ± 0.042^a^	0.463 ± 0.132^a^	0.006 ± 0.002^a^	35.017 ± 2.381^d^	0.518 ± 0.324^a^	6.287 ± 0.210^d^
FHXK2	0.144 ± 0.026^a^	0.553 ± 0.054^a^	0.007 ± 0.000^a^	48.212 ± 2.214^e^	0.482 ± 0.054^a^	7.380 ± 0.289^e^
FPYK1	0.145 ± 0.019^a^	0.409 ± 0.175^a^	0.009 ± 0.001^a^	26.628 ± 0.275^c^	0.599 ± 0.041^a^	7.100 ± 0.170^e^
FREG1	0.176 ± 0.068^a^	0.129 ± 0.029^a^	0.154 ± 0.021^b^	10.594 ± 2.920^a^	0.973 ± 0.119^a^	1.273 ± 0.178^a^
FPDC1	0.130 ± 0.034^a^	1.875 ± 0.478^b^	0.005 ± 0.001^a^	15.557 ± 1.273^b^	0.391 ± 0.069^a^	2.244 ± 0.173^b^
FPDE2	0.125 ± 0.039^a^	0.389 ± 0.055^a^	0.020 ± 0.005^a^	31.113 ± 1.961^cd^	1.943 ± 0.549^b^	4.570 ± 0.500^c^

Values are shown as mean of relative abundance on the internal standard ± SD of at least two biological replicates, after 7 days of fermentation. Different letters indicate statistically significant differences (HSD Tukey) for values in the same column

The relative similarity in acetaldehyde production shown by the yeast strains under anaerobic conditions does not translate into a similar uniformity concerning acetaldehyde derived products. Anaerobic cultures of FREG1 and FPDE2 showed a clear increase in acetoin (25-fold or four-fold respectively) and 2,3 butanediol (two-fold increase and four-fold) levels, as compared to FX10 (Table 4). Following the trend seen for acetaldehyde, all the strains produced clearly increased amounts of acetoin or 2,3 butanediol under aerobic conditions. Again, the behavior of strains deleted for *REG1* or *PDC1* was clearly different from other yeast strains. FREG1 and FPDC1 showed the lowest values of both acetoin and 2,3 butanediol in aerobiosis (Table 4). In addition, the difference between aerobic and anaerobic conditions observed for FREG1, concerning the content of these two compounds was the smallest among all the strains analyzed.

**Fig. 3:**
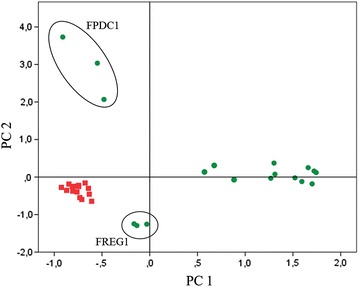
Principal component analysis based on the yields of main fermentation products and pyruvate derived metabolites. The PC1 and PC2 explained 64.89 and 81.97 % respectively of the total yeast strains variance under aerobic (*green dots*) and anaerobic (*red squares*) conditions

Finally, we performed a principal component analysis by taking into account ethanol, acetic acid, and glycerol, as well as acetaldehyde, acetoin, and 2,3 butanediol. The results confirm the important metabolic differences between aerobic and anaerobic cultures (Fig. 3). Samples were clearly separated along the PC1 axis, depending on the oxygenation conditions, with anaerobic samples showing lower production of acetoin, 2,3 butanediol, and acetic acid; and high production of ethanol and glycerol, as already seen in Tables 3 and 4. However, two strains, FREG1 and FPDC1 showed, under aerobic conditions, a behavior reminiscent of anaerobic cultures. This was clear for acetic acid yield and 2,3 butanediol production in the case of FREG1, and also evident for FPDC1 concerning acetic acid yield, as well as acetoin and 2,3 butanediol production. Aerobic samples of FPDC1 were clearly separated from the rest of the samples along the PC2 axis, mostly due to the high acetaldehyde production of this strain in the presence of oxygen. The high production of glycerol by FREG1 under aerobic conditions explains the low position of these samples along the PC2 axis.

## Discussion

Despite the Crabtree effect, there was a huge impact of oxygen availability on yeast metabolism for all the strains assayed in this work. Indeed, reduced ethanol yield in the aerobic fermentation of natural or synthetic grape must was already described for *S. cerevisiae* in our previous work [9, 11]. This indicates a relevant portion of the sugar was metabolized by respiration under aerobic conditions, a conclusion that is also sustained by an important increase in biomass yield in aerobic cultures. For most of the yeast strains studied, production of other metabolites was also highly affected by culture under aerobic conditions, as compared to anaerobiosis, including acetic acid and glycerol, also in agreement with previous publications [10, 25]; as well as other pyruvate derived metabolites, like acetaldehyde, acetoin or 2,3 butanediol.

A clear alleviation of the Crabtree effect was previously described for loss-of-function mutations in several of the genes studied in this work, including *HXK2*, *PYK1* and *REG1* [15, 20]. Surprisingly, we found little or no impact of these gene deletions on ethanol or biomass yield in the FX10 genetic background under our experimental conditions. Claims on the relief of the Crabtree effect for these gene deletions are mostly based on chemostat cultures under carbon limited conditions. In contrast, natural grape must contains limiting amounts of yeast assimilable nitrogen, while carbon sources (glucose and fructose) are in great excess. Our results illustrate the impact of the cultivation mode in order to assess yeast metabolic features. In this way, genetic modification resulting in an important change in the critical dilution rate under chemostat growing conditions might appear as almost irrelevant for cultures in batch, especially for growth in high sugar content media. Several authors postulate that the Crabtree effect in *S. cerevisiae* is mostly a manifestation of an overflow metabolism at the level of pyruvate [13, 14]. Indeed, a total relief of the Crabtree effect in this species has only been attained by an almost complete impairment of glucose intake by the cells [30]. According to this model, some gene deletions affecting the glycolytic rate (i.e. the rate of production of pyruvate), or the capacities of enzymes involved in further pyruvate metabolism, might have a clear impact on the critical dilution rate [15, 20]. However, the extreme overflow we can expect for batch cultures with around 200 g/L initial sugar content might be almost insensitive to the same gene modifications. This is exactly what we observed for some of the gene modifications initially selected in this work.

Despite the low impact of the assayed gene modifications on ethanol yields under aerobic conditions, a clear reduction in ethanol levels, as compared to anaerobic conditions, was observed for all strains. One of the problems associated with aeration during wine fermentation is increased acetic acid production, as shown in Table 2 and as already observed in previous works [9–11]. Our preliminary results showed that, despite not being intended for that purpose, some gene modifications seemed to result in clearly reduced volatile acidity. The practical implications of this finding prompted us to include some additional genes in the study.

According to results in other genetic backgrounds [15, 18, 20], deletion of *PYK1* (hemizygous) or *HXK2* in FX10 results in glucose derepression. Other authors have described low ethanol yield in aerobic batch cultures of strains deleted for *HXK2*, or showing reduced levels of pyruvate kinase activity [16, 20]. However, the behavior of FHXK2, FPYK1 and FX10 strains in this work was almost identical, only minor (although statistically significant) differences in metabolic footprint were found for acetoin and 2,3 butanediol production, and only for aerobic cultures. The low impact of these gene deletions during the fermentation of natural grape must is thus in contrast with the results by Diderich et al. [16] and Pierce et al. [20]. There are at least two non-exclusive explanations to this discrepancy. One is based in media composition. Initial glucose content in the batch cultures by the later authors ranged from 10 to 20 g/L, while our natural grape must contain about 200 g/L (equimolar amounts of glucose and fructose). In addition, yeast assimilable nitrogen level in grape must is low, so that most of the sugar is metabolized under nitrogen limitation. This is in contrast with synthetic media for which nitrogen sources were in excess. A second explanation in the case of *PYK1* deletion is FPYK1 was hemizygous for that deletion (*PYK1* is an essential yeast gene). The maximal reduction in pyruvate kinase activity we would expect from this construction is 50 %. In contrast, Pierce et al. [20] used a construction resulting in a reduction of pyruvate kinase levels down to 20–25 % of normal values. The high similarity of FX10, FHXK2 and FPYK1 under our experimental conditions, either aerobic or anaerobic, despite the different behavior shown under chemostat conditions [15, 20] is illustrative of the lack of predictive power of standard Crabtree assays for certain industrially relevant conditions, as discussed above.

**Fig. 4:**
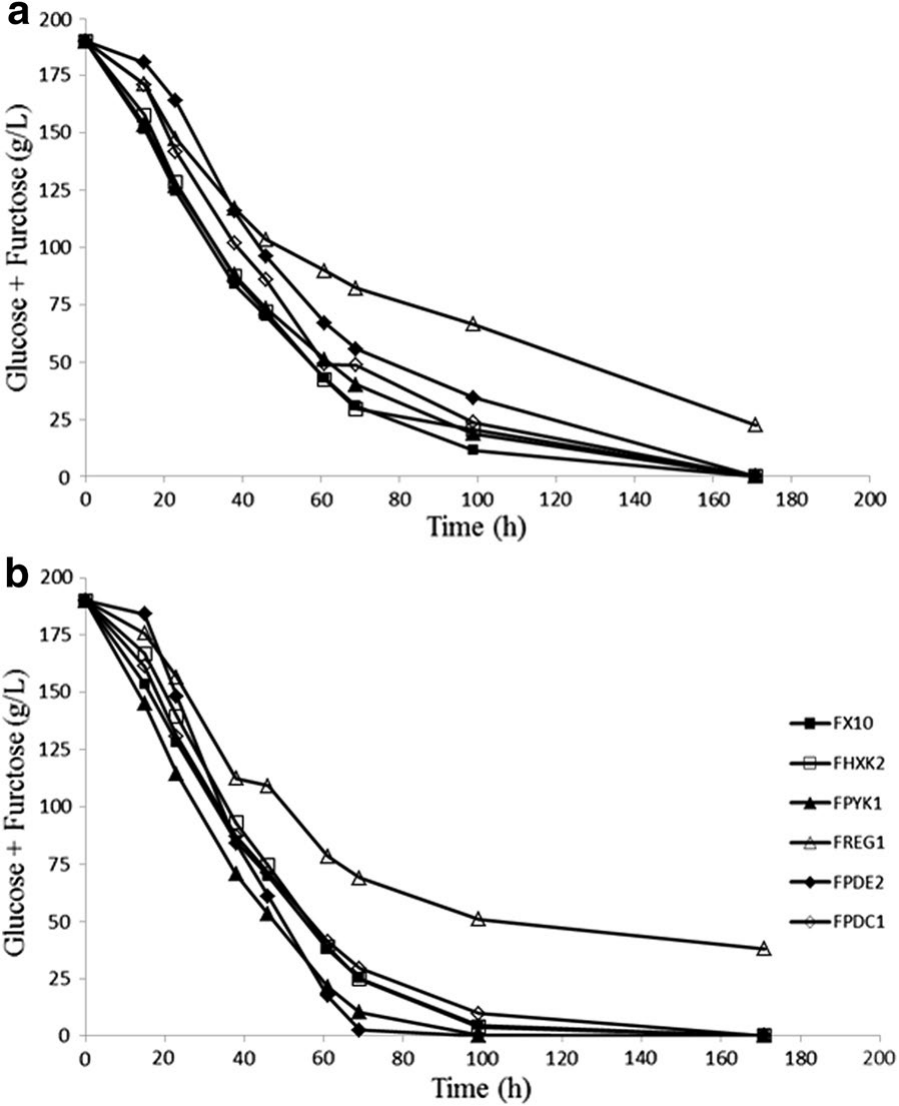
Sugar consumption kinetics during anaerobic (**a**) and aerobic (**b**) fermentations of the strains indicated. Results are the average of biological triplicates Please, refer to Additional file 2: Figure S2 for SD (removed in this graph for clarity)

Strain FPDE2 shows the lowest biomass production under both aerobic and anaerobic growth conditions, despite it is able to reach complete fermentation of grape must with a kinetics similar to FX10 (Fig. 4). Since the cyclic AMP phosphodiesterase encoded by *PDE2* is involved in reducing cAMP levels, and despite it can be partially substituted by Pde1p [31], we would expect cell functions regulated by PKA to be altered FPDE2 [32]. Two opposite effects on biomass production would be expected. By one side, increased PKA activity would involve activation of glycolysis, growth, and proliferation. By the other side, stress response would be reduced, resulting in low tolerance to the harsh conditions in grape must, and notably osmotic stress. Our results indicate the later effect would be dominant and result in the low biomass of FPDE2 cultures under both aerobic and anaerobic conditions. The expected positive impact of the deletion of *PDE2* on glycolysis rate might exacerbate overflow metabolism and be responsible for the fact that FPDE2 appears in the upper part of the distribution of ethanol yield under aerobic conditions, being the strain showing the minor reduction in ethanol yield from anaerobic to aerobic fermentation conditions. In addition to ethanol, this gene deletion has a limited impact on the yields of acetic acid, glycerol and 2,3 butanediol in anaerobiosis; as well as on acetic acid yield in aerobic fermentations. In practical terms, the reduction of acetic acid yield under aerobic conditions by FPDE2 might have an advantage over FX10, in order to attain alcohol level reduction by aeration of the fermenting must. However, other mutant strains assayed in this work seem to be more interesting for the development of industrial strains (see below).

*PDC1* codes for the main pyruvate decarboxylase isozyme in *S. cerevisiae*. Deletion of this gene in the FX10 background resulted in little impact under anaerobic conditions, but it had relevant consequences for aerobic fermentation. Under these conditions, the ethanol yield of FDC1 was similar to the control strain, but it showed a clear reduction in acetic acid yield, as well as acetoin and 2,3 butanediol. In contrast, a relevant increase in acetaldehyde production was observed. Although the main activity of Pdc1 is the conversion of pyruvate to acetaldehyde, acetoin has been described as one main side product of the reaction. Acetoin can in turn be transformed to 2,3 butanediol. It has been shown that Pdc5, the other major pyruvate decarboxylase isozyme, is able to warrant about 70 % of the pyruvate decarboxylase activity required by the cell in *∆pdc1* strains [33]. However, our results suggest differences in acetoin production between Pdc1 and Pdc5, resulting in acetaldehyde accumulation at the expense of acetoin and 2,3 butanediol. Perhaps the most interesting feature of FPDC1 concerning its application in wine making is the low acetic acid yield in aerobic fermentation, resulting in volatile acidity values around the 0.8 g/L threshold.

Deletion of *REG1* is probably the most pleiotropic gene modification among those assayed in this work. This was to be expected, given the upstream position of Reg1 in the glucose sensing signal transduction pathway (Fig. 1). In addition, the substrate of the GLC7-Reg1 protein phosphatase complex is Snf1, and this protein kinase is involved in the regulation of many cellular functions. Concerning anaerobic conditions, FREG1 was almost the only strain showing statistically significant differences with the control strain for ethanol yield or acetoin production. However, the most interesting impact of this gene modification was observed for aerobic cultures. By one side, it showed the lower ethanol yield values among the yeast strains used in this work. More interesting is the fact that this strain also showed the lower values for acetic acid yield under aerobic conditions, as well as for acetaldehyde, acetoin or 2,3 butanediol. In contrast to all the other yeast strains, almost no difference in acetic acid yield was observed for this strain between aerobic and anaerobic fermentations, and those values were similar to anaerobic cultures of FX10. Also relevant is the increase in the aerobic glycerol yield for this strain as compared to anaerobic growth (about two-times). This was indeed opposite to the other strains, showing a two-fold decrease in glycerol yield for the same growth conditions. This result seems to be in contrast with reports showing an increase in the production of acetic acid for genetic modifications aiming to glycerol overproduction [34]. However, these reports are based on growth under standard fermentation conditions while glycerol overproduction by FREG1 without an increase in acetic acid production takes place under aerobic fermentation conditions. Obviously, the redox compensation mechanisms involved in linking acetic acid and glycerol metabolism under anaerobic conditions are not operating the same way in the presence of oxygen. An interesting feature of *REG1* loss-of-function yeast mutants, is they can be easily obtained and selected by random mutagenesis [35, 36]. This opens the way for obtaining non-GMO wine yeast strains similar to FREG1, which would be readily available for winemakers, avoiding the limitations associated to recombinant wine yeasts [37]. The hurdle imposed by the recessive character of *REG1* defective mutants can be overcome by sporulating yeast strains before or after random mutagenesis. The use of homozygous wine yeast strains, like FX10, is advisable for this approach.

## Conclusions

We report the metabolic characterization of five gene deletions in a commercial *S. cerevisiae* yeast background during anaerobic and aerobic fermentation of natural grape must. Oxygenation of grape must is sufficient to warrant a relevant reduction in final ethanol content. The impact on ethanol yields of the gene deletions assayed in this work was negligible, both under aerobic and anaerobic fermentation conditions. However, some of these deletions did contribute to solve the main drawback of aerobic fermentation concerning winemaking, excess acetic acid production (resulting in high volatile acidity). The most promising results from this point of view were shown by the carbon catabolite derepressed strain FREG1, deleted for *REG1*. Similar strains would be easily obtained by classical genetic techniques. Such strains, in combination with a wild type strain, would be useful for the commercial production of wines with reduced ethanol content.

## Methods

### Strains and growth conditions

*Saccharomyces cerevisiae* Zymaflore® FX10 (Laffort), a homozygous and homothallic commercial wine yeast strain was used as host strain for genetic modification. The heterozygous mutants were constructed using the short flanking homology method [38], by transforming FX10 using the lithium acetate procedure [39] with a PCR fragment obtained by amplification of the kanMX4 cassette and flanking regions from the appropriate homozygous deletion strain in the BY4743 background (Open Biosystems, Huntsville, USA). Selection of heterozygous mutants was performed in YPD solid media plates supplemented with 200 mg/L of geneticin (G418). Correct insertion of the kanMX4 cassette was verified by PCR using primers upstream and downstream of the deleted region combined with primers inside kanMX4. Primers used for the construction and verification of recombinant strains are shown in Additional file 3: Table S1. Amplification strategies are summarized in Additional file 4: Figure S3. The homozygous mutants were constructed by sporulating the heterozygous mutants in a medium supplemented with geneticin. The geneticin resistance feature segregated 2:2 as expected. Since the original strain was homothallic, strains recovered from the segregation analysis plates were spontaneous autodiploids, homozygous for the corresponding gene deletion, as verified by PCR (see above).

Yeast strains were grown at 28 °C and maintained at 4 °C on yeast peptone dextrose (YPD) plates (2 % glucose, 2 % peptone, 1 % yeast extract, and 2 % agar), as well as in glycerol stocks at −80 °C. Fermentation experiments were performed in natural grape must (ca. 200 g/L sugars).

### Fermentation assays

Batch fermentation were carried out in triplicate in bioreactors equipped with refrigerated gas condensers (Dasgip, Eppendorf, Germany). Bioreactors were filled with 250 mL of natural must and sparged at a gas flow rate of 2.5 L/h with either air or nitrogen. Gas flow was controlled with MFC17 mass flow controller (Aalborg Instruments and Controls, Inc., Orangeburg, NY), whose calibration was regularly verified with automatic flowmeters. Temperature was set to 28 °C, stirring to 200 rpm and inoculation to approximately 0.2 initial optical density at 600 nm (OD_600_). pH was continuously adjusted to 3.5 during fermentation progress by the automatic addition of 2 M NaOH.

### *2*-Deoxy glucose sensitivity assay

Yeast strains were spotted at different dilutions (10^−1^ to 10^−4^) on YP plates (10 g/L yeast extract and 20 g/L Bacto peptone) that contained 2 % of galactose as carbon source, supplemented with 200 µg/mL of 2-deoxy glucose. Plates were incubated for 48 h at 28 °C.

### Quantification of main fermentation-related metabolites

Production or consumption of glucose, fructose, glycerol, acetic acid and ethanol, were determined by HPLC in duplicate, using a Surveyor Plus chromatograph (Thermo Fisher Scientific, Waltham, MA) equipped with a refraction index and a photodiode array detector (Surveyor RI Plus and Surveyor PDA Plus, respectively). A Hyper REZ XP carbohydrate H+ 8 μm column and guard (Thermo Fisher Scientific) were used and maintained at 50 °C. Elution was performed with 1.5 mM H_2_SO_4_ as mobile phase, at a flow rate of 0.6 mL/min. Prior to injection, samples were filtered through 0.22-μm-pore-size nylon filters and diluted 10-fold.

### Analysis of volatile compounds

500 µL of sample were placed in a 2 mL glass-vial with 1 mL of ammonium sulphate solution (45 % w/v) and extracted with 250 µL of methyl acetate-ethanol solution (99.5:0.5, v/v) containing 50 ppm of internal standards (4-methyl 2-pentanol, 1-nonanol, and heptanoic acid). A 3 µL sample of the upper, methyl acetate phase, was injected with the SSL liner held at 180 °C.

Gas chromatography–mass spectrometry was carried out in a Thermo TRACE GC Ultra apparatus equipped with a Thermo TriPlus autosampler with a fused-silica capillary column TG-WAXMS A (30 m long; 0.25 mm OD; 0.25 µm film thickness) coupled to a Thermo ISQ mass detector.

Chromatographic conditions were as follows: 5 min at 40 °C, 3 °C/min up to 200 °C, 15 °C/min up to 240 °C, and 10 min at 240 °C. Helium was used as carrier gas at a flow rate of 1 mL/min, operating in split mode (ratio 30). Detection was performed with the mass spectrometer operating in the Full Scan mode (dwell time 500 ms), with 70 eV ionization energy, and source and quadrupole temperatures of 250 °C. Peaks were identified by comparison of retention times and ion spectra from real standards (Sigma-Aldrich Química) and spectra from the NIST mass spectral library. For each compound, including internal standards, the sum of the areas of the peaks of up to five characteristic ions was obtained.

### Statistical analysis

One way analysis of variance was carried out on the main fermentation metabolites found on day 7. Average of biological triplicates was compared using Tukey’s test, with significance level set at 5 %. All analyses were performed using SPSS Statistics v. 20 program (IBM, Armonk, NY).

## Additional files

10.1186/s12934-016-0555-y Schematic representation of the pyruvate node in* S. cerevisiae*. Background arrows indicate carbon flux distribution under excess sugar, aerobic conditions for Crabtree-positive yeasts (overflow metabolism at the pyruvate node level). Some of the genes deleted in this work (underlined) are shown; as well as genes coding for cognate isoenzymes.* PEP* phosphoenolpyruvate.
10.1186/s12934-016-0555-y Kinetics of glucose and fructose consumption under aerobic (A) or anaerobic (B) conditions. Results are the average of biological triplicates. Error bars correspond to ±SD from three biological replicates.
10.1186/s12934-016-0555-y Primers used in this study.
10.1186/s12934-016-0555-y Summary of all PCR reactions performed in this work. In black, ORFs to be replaced in FX10. They were replaced by the kanMX4 cassette (shown in the bottom of the figure) by amplifying the whole region from the appropriate homozygous deletion strain in the BY4743 background, and using it to transform FX10. Primers with the “KO” label (see figure) were used for this purpose. Correct insertion was verified by using primers with the “OUT” label, in single and double FX10 deletion strains. In addition, in order to avoid ambiguities, additional confirmation PCR reactions were run by using one of the “OUT” primers from each pair and the “kanMX4 IN R” primer. In this case, additional control PCR reactions were run with the “IN” labelled primers (see figure). All PCR verification reactions were run in parallel with genomic DNA from the parent and the putative recombinant strain.
