# Supplementary figures and images for: Identification of target genes to control acetate yield during aerobic fermentation with Saccharomyces cerevisiae
Source: Microb Cell Fact. 2016 Sep 15;15:156. doi: 10.1186/s12934-016-0555-y (PMC5024518; doi:10.1186/s12934-016-0555-y)

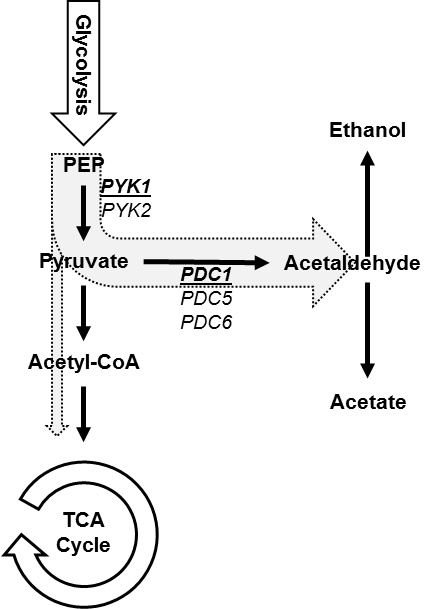

Supplement: Supplementary file 1 — 10.1186/s12934-016-0555-y Schematic representation of the pyruvate node in S. cerevisiae. Background arrows indicate carbon flux distribution under excess sugar, aerobic conditions for Crabtree-positive yeasts (overflow metabolism at the pyruvate node level). Some of the genes deleted in this work (underlined) are shown; as well as genes coding for cognate isoenzymes. PEP phosphoenolpyruvate. [file 12934_2016_555_MOESM1_ESM.tif]

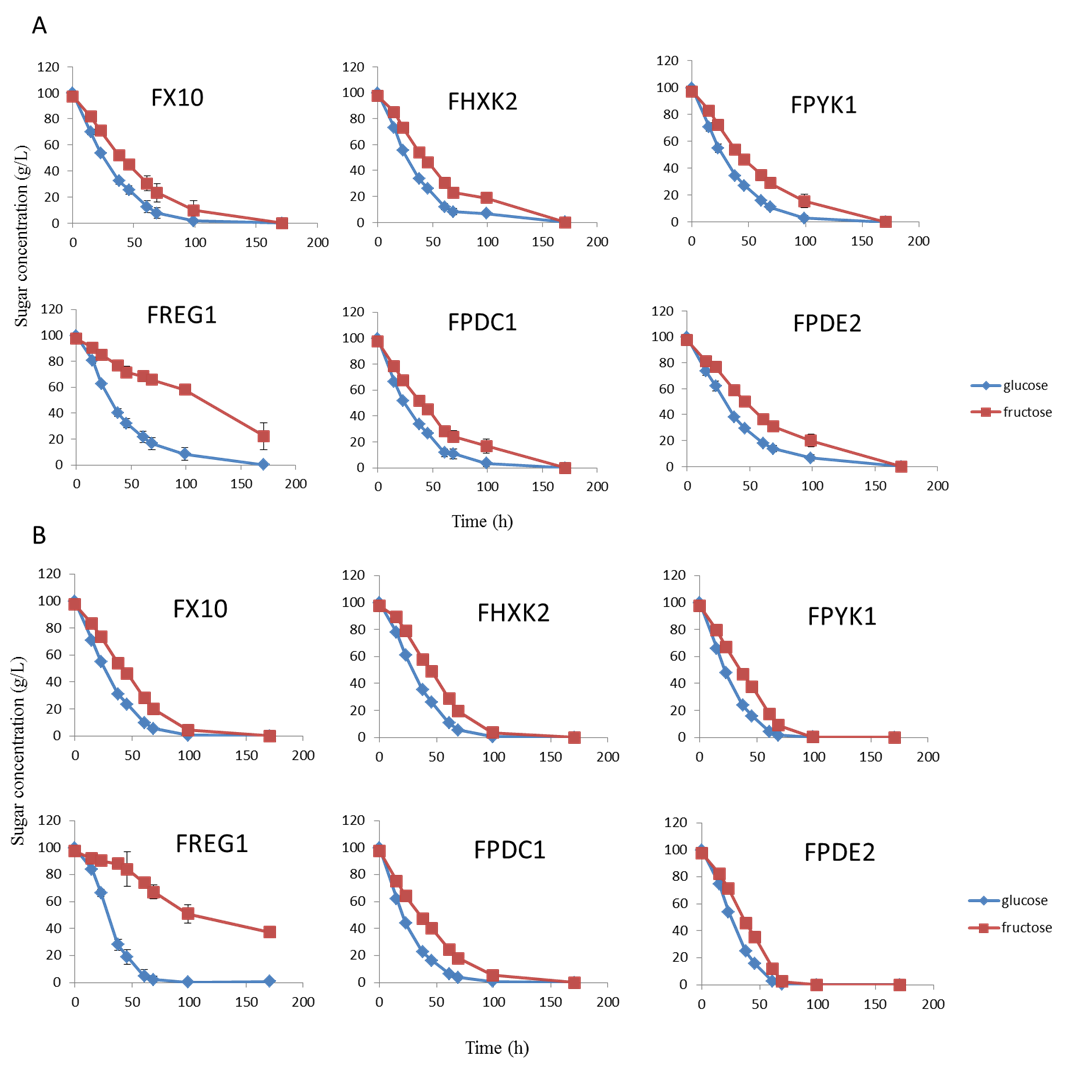

Supplement: Supplementary file 2 — 10.1186/s12934-016-0555-y Kinetics of glucose and fructose consumption under aerobic (A) or anaerobic (B) conditions. Results are the average of biological triplicates. Error bars correspond to ±SD from three biological replicates. [file 12934_2016_555_MOESM2_ESM.tif]

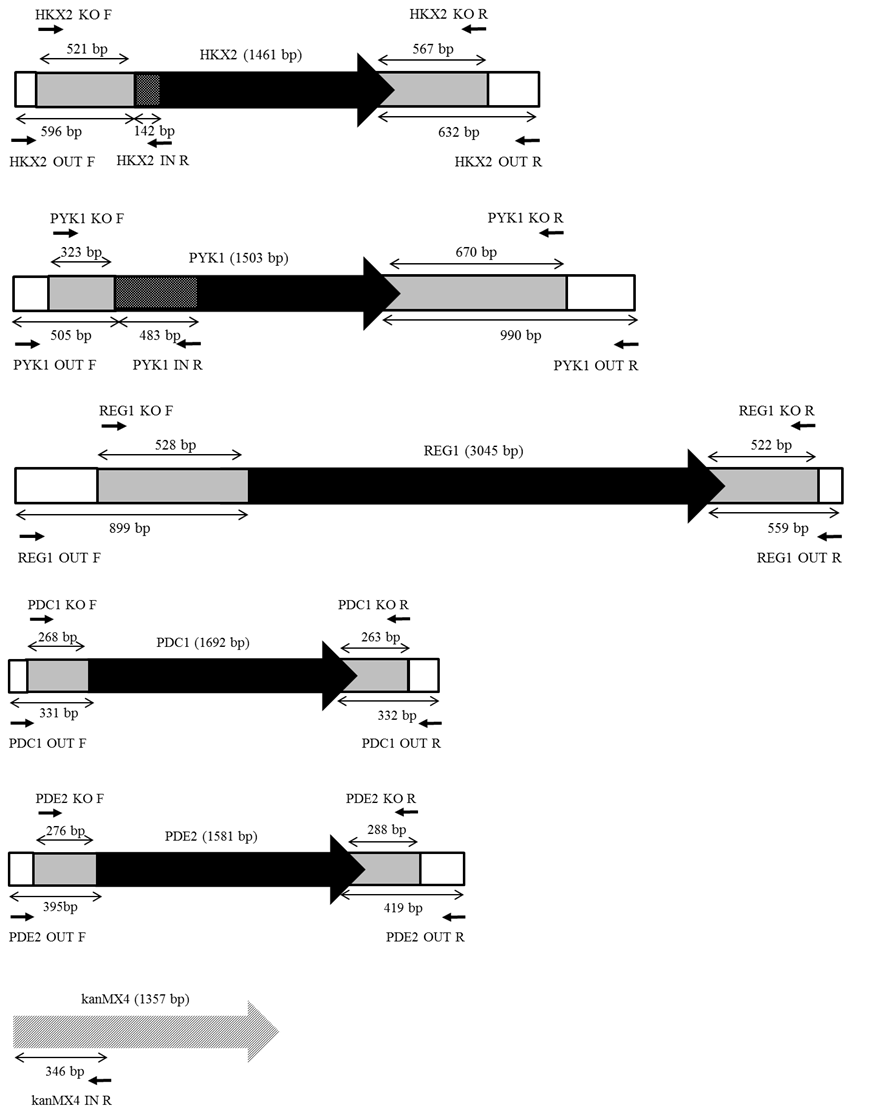

Supplement: Supplementary file 4 — 10.1186/s12934-016-0555-y Summary of all PCR reactions performed in this work. In black, ORFs to be replaced in FX10. They were replaced by the kanMX4 cassette (shown in the bottom of the figure) by amplifying the whole region from the appropriate homozygous deletion strain in the BY4743 background, and using it to transform FX10. Primers with the “KO” label (see figure) were used for this purpose. Correct insertion was verified by using primers with the “OUT” label, in single and double FX10 deletion strains. In addition, in order to avoid ambiguities, additional confirmation PCR reactions were run by using one of the “OUT” primers from each pair and the “kanMX4 IN R” primer. In this case, additional control PCR reactions were run with the “IN” labelled primers (see figure). All PCR verification reactions were run in parallel with genomic DNA from the parent and the putative recombinant strain. [file 12934_2016_555_MOESM4_ESM.tif]
