# Supplementary material for: Identification of target genes to control acetate yield during aerobic fermentation with Saccharomyces cerevisiae
Source: Microb Cell Fact. 2016 Sep 15;15:156. doi: 10.1186/s12934-016-0555-y (PMC5024518; doi:10.1186/s12934-016-0555-y)
Supplement: Supplementary file 3 — 10.1186/s12934-016-0555-y Primers used in this study. [file 12934_2016_555_MOESM3_ESM.docx]

Table 1S. Primers used in this study.

| Primer | Sequence (5’ 🡪 3’) |
| --- | --- |
| HXK2 KO F | TTCTGAACCTCCTCGCACAT |
| HXK2 KO R | AGCGTAGTGAGGTGGAGACC |
| HXK2 OUT F | CTCCAGAGCTCCACATTGGT |
| HXK2 OUT R | TGTGATTTGCGGTGTTCATC |
| HXK2 IN R | AGTGCTTGGTAACGGCTTGT |
| PYK1 KO F | GCTTGTGATGTCTTCCAAGTGA |
| PYK1 KO R | CCACTTCAGTTTTCTTCCCATCT |
| PYK1 OUT F | CCATCGACAGATTGGGAGAT |
| PYK1 OUT R | CGGCCTTCTTTGTTGCTACT |
| PYK1 IN R | CTTGTCGTCAACGACTTCCA |
| REG1 KO F | GGATATTGAAGGAAGGAATCAGC |
| REG1 KO R | TTGACATTGGCCAGATACCTC |
| REG1 OUT F | GCGGATCCATCTTTGAATGT |
| REG1 OUT R | TGCAGTCCCTGGCTTTTATT |
| PDC1 KO F | CCTTGGTTCCACTAATTCATCGG |
| PDC1 KO R | TGCTATCGTTCAACACCACCT |
| PDC1 OUT F | TATTGTCCGCTGCCCCTTTT |
| PDC1 OUT R | TCAGGGTTTTGGAAACCACAC |
| PDE2 KO F | ACTCGGGAAATATGTATCACTAT |
| PDE2 KO R | TGGCTTAGAGAGAATATACTTGC |
| PDE2 OUT F | AAGGGTCCTGCGTCCTTTTC |
| PDE2 OUT R | TGCATATACCAACACAGGGAACA |
| kanMX4 IN R | CTGCAGCGAGGAGCCGTAAT |
